# Factors Associated with Systemic Bleeding in *Bothrops* Envenomation in a Tertiary Hospital in the Brazilian Amazon

**DOI:** 10.3390/toxins11010022

**Published:** 2019-01-07

**Authors:** Sâmella S. Oliveira, Eliane C. Alves, Alessandra S. Santos, João Pedro T. Pereira, Lybia Kássia S. Sarraff, Elizandra F. Nascimento, José Diego de-Brito-Sousa, Vanderson S. Sampaio, Marcus V.G. Lacerda, Jacqueline A.G. Sachett, Ida S. Sano-Martins, Wuelton M. Monteiro

**Affiliations:** 1 Escola Superior de Ciências da Saúde, Universidade do Estado do Amazonas, Manaus 69065-001, Brazil; oliveira.samella@gmail.com (S.S.O.); anealves.enf@gmail.com (E.C.A.); sousajdb@live.com (J.D.d.-B.-S.); vandersons@gmail.com (V.S.S.); jac.sachett@gmail.com (J.A.G.S.); 2 Instituto de Pesquisa Clínica Carlos Borborema, Fundação de Medicina Tropical Dr. Heitor Vieira Dourado, Manaus 69040-000, Brazil; alessandrasantos910@gmail.com (A.S.S.); pedro02.tavares@gmail.com (J.P.T.P.); lybsarraff@gmail.com (L.K.S.S.); elyfreitas46@gmail.com (E.F.N.); marcuslacerda.br@gmail.com (M.V.G.L.); 3 Laboratório de Fisiopatologia, Instituto Butantan, São Paulo 05503-900, Brazil; ida.sano@butantan.gov.br

**Keywords:** hemostatic disorders, unclottable blood, thrombocytopenia, *Bothrops atrox*

## Abstract

*Bothrops* snakebites usually present systemic bleeding, and the clinical–epidemiological and laboratorial factors associated with the development of this manifestation are not well established. In this study, we assessed the prevalence of *Bothrops* snakebites with systemic bleeding reported at the *Fundação de Medicina Tropical Dr. Heitor Vieira Dourado*, in Manaus, Amazonas State, Brazil, and the clinical–epidemiological and laboratorial factors associated with systemic bleeding. This is an observational, cross-sectional study carried out between August, 2013 and July, 2016. Patients who developed systemic bleeding on admission or during hospitalization were considered cases, and those with non-systemic bleeding were included in the control group. Systemic bleeding was observed in 63 (15.3%) of the 442 *Bothrops* snakebites evaluated. *Bothrops* snakebites mostly occurred in males (78.2%), in rural areas (89.0%) and in the age group of 11 to 30 years old (40.4%). It took most of the patients (59.8%) less than 3 h to receive medical assistance. Unclottable blood (AOR = 3.11 (95% CI = 1.53 to 6.31; *p* = 0.002)) and thrombocytopenia (AOR = 4.52 (95% CI = 2.03 to 10.09; *p* < 0.001)) on admission were independently associated with systemic bleeding during hospitalization. These hemostatic disorders on admission increase the chances of systemic bleeding during hospitalization. Prospective studies are needed to clarify the pathophysiology of systemic bleeding in *Bothrops* snakebites in the Amazon region.

## 1. Introduction

Snakebite envenomation is a potentially life-threatening injury and a major public health problem in rural areas of tropical and sub-tropical countries of Africa, the Middle-East, Asia, Oceania, and Latin America. Global estimates suggest that at least 400,000 snakebites occur annually, which result in 20,000 deaths [1]. In Brazil, approximately 26,000 snakebites of medical importance are reported each year, with an average lethality rate of 0.4% [2]. The Amazon region reports the highest incidence of snakebite envenomation in Brazil, with 52.6 cases/100,000 inhabitants [3]. In the Amazonas State, an incidence rate of 52.8 cases/100,000 inhabitants and a 0.6% lethality rate can be observed [4]. Injuries occurring at a distance >300 Km from Manaus, the Amazonas State capital, the age of the victim ≥61 years, and indigenous status were factors that were independently associated with case fatality from snakebites [5]. The *Bothrops* genus is responsible for the highest frequency of snakebites in the Brazilian Amazon, causing 80% to 90% of the cases in the region [6]. *Bothrops atrox* is the main species of snake involved in these snakebites [6,7]. *Bothrops* snakebite victims usually present local symptoms (i.e. pain, swelling, ecchymosis, and blistering) and systemic manifestations such as bleeding and coagulation disorders [7,8,9]. Complications such as necrosis, secondary bacterial infection, compartment syndrome, and acute renal failure may also occur [8]. Although cases of death are rare, they are frequently associated with renal and respiratory failure, shock, sepsis, and hemorrhage in the central nervous system [9]. In fatal cases of *Bothrops* snakebites in the Amazonas State, systemic bleeding, circulatory shock, sepsis, and acute respiratory failure were usually observed [5]. One case of fatal hemorrhagic stroke after a *Bothrops* snakebite in the Amazonas State, in which the presence of venom in the patient’s brain tissue was identified after death, has been reported. The victim received *Bothrops* antivenom two and a half hours after the bite [10].

Clinical manifestations observed in *Bothrops* snakebites appear as result of the action of the snake venom [11]. The components that are most abundant in *B. atrox* venom are snake venom metalloproteinases (SVMPs), snake venom serine proteinases (SVSPs), and phospholipases A_2_ (PLA_2_) [12]. These components of the snake venom can affect hemostasis by activating or inhibiting coagulant factors or platelets, or by disrupting endothelium or by causing thrombosis [13,14]. The association of the coagulant action of venom, with its activity on platelets and vascular endothelium, could lead to a higher incidence of systemic bleeding as a result of the snakebite [15]. Hemodynamic changes, which may result in cardiovascular shock, may occur as a consequence of systemic bleeding [16]. However, the composition and biological activities of snake venoms may vary, not only between different species of snakes (interspecific variation), but also within the same species (intraspecific variation) depending on the geographical origin, ontogenetic stage, habitat, or sex of the snake, thus influencing the clinical manifestations of snakebites [17,18].

It has been observed that between 7% and 53% of *Bothrops* snakebites develop systemic bleeding [7,15,19,20,21]. It is not clear why some *Bothrops* snakebite patients develop systemic bleeding and others do not. A better understanding of the development of systemic bleeding in patients who have suffered *Bothrops* snakebites would lead to improved management and understanding of the pathophysiology of envenomation of snakes that have medical importance in the Brazilian Amazon. Thus, the aim of this study was to assess the prevalence of *Bothrops* snakebites in which systemic bleeding was reported at a specialist hospital for snakebites in Manaus, Amazonas State, and the clinical–epidemiological and laboratorial factors associated with systemic bleeding in victims of envenomation by *Bothrops* snakes. The results show that systemic bleeding observed in *Bothrops* snakebite patients is an important systemic effect of the envenoming. Thrombocytopenia and unclottable blood detected on admission are factors associated with systemic bleeding during hospitalization. Moreover, the effect of other parameters, such as some toxins components of the venom, cannot be ruled out.

## 2. Results

### 2.1. Study Population

**Figure 1:**
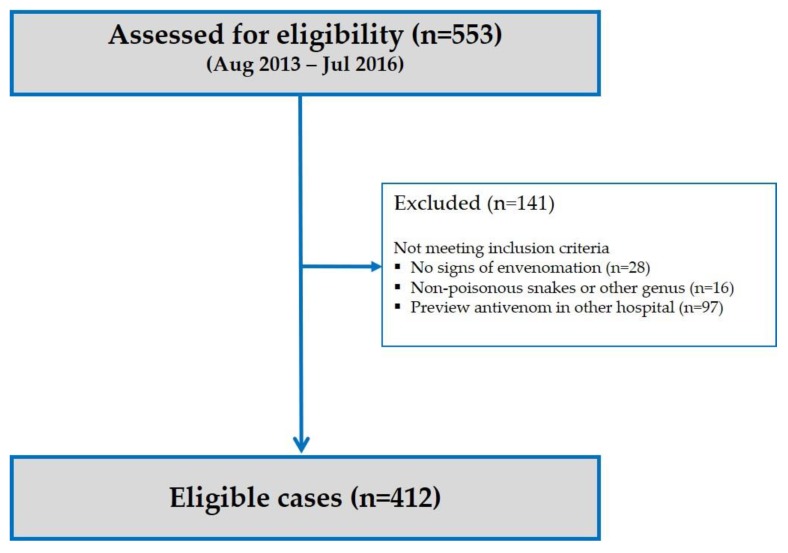
Flow chart for the inclusion of patient with clinical–epidemiological diagnosis of *Bothrops* envenomation in our study.

A total of 553 patients with clinical–epidemiological diagnosis of *Bothrops* envenomation were assessed for eligibility over the three year study period. From this total, 412 were eligible for inclusion in our study (Figure 1). The majority of *Bothrops* envenomations were in males (78.2%) and in the age group of 11 to 30 years old (40.4%). These snakebites mostly occurred in Manaus (57.4%), Amazonas State capital, in rural areas (89.0%), and only 39.8% were related to work. The majority of patients (59.8%) took less than 3 h to reach medical assistance. The most affected anatomical site was the foot (72.1%). The *Bothrops* bite was confirmed by snake identification in 32.8% of the included patients’ records. All *Bothrops* snakes were identified as being *Bothrops atrox* (Table 1).

Clinical characterization of *Bothrops* snakebite patients on admission showed that the most frequent local manifestations were pain (93.9%) followed by swelling (93.4%). Among the systemic alterations related to hemostasis, unclottable blood (58.2%), bleeding (12.6%), and thrombocytopenia (8.7%) were observed. Other common systemic manifestations were headache (11.4%), nausea (10.7%), and vomiting (9.7%). Most cases were moderate (54.2%), followed by mild (27.7%) cases. Comorbidities such as arterial hypertension (9.3%) and diabetes (2.6%) were also observed. There were no recorded deaths due to a snakebite in this study period (Table 2).

### 2.2. Hemostasis Parameters

Systemic bleeding was observed during hospitalization in 15.3% of the *Bothrops* snakebite patients. Conjunctival bleeding was the most frequent form of systemic bleeding (6.7%), followed by gingival bleeding (6.3%) (Table 3). The majority of the *Bothrops* snakebite patients with systemic bleeding during hospitalization showed a platelet count on admission that was higher than 150,000/µL, irrespective of the type of systemic bleeding, except for one patient who presented petechiae (<50,000/µL) (Table 4).

**Figure 2:**
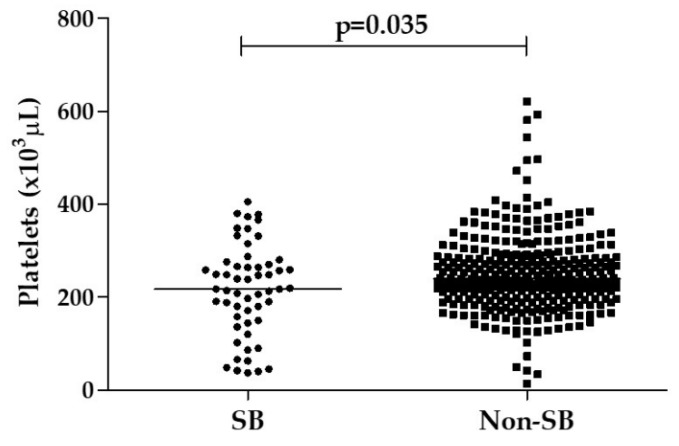
Platelet counts obtained on admission for *Bothrops* snakebite patients that showed systemic bleeding (SB) or not (Non-SB) during hospitalization.

**Figure 3:**
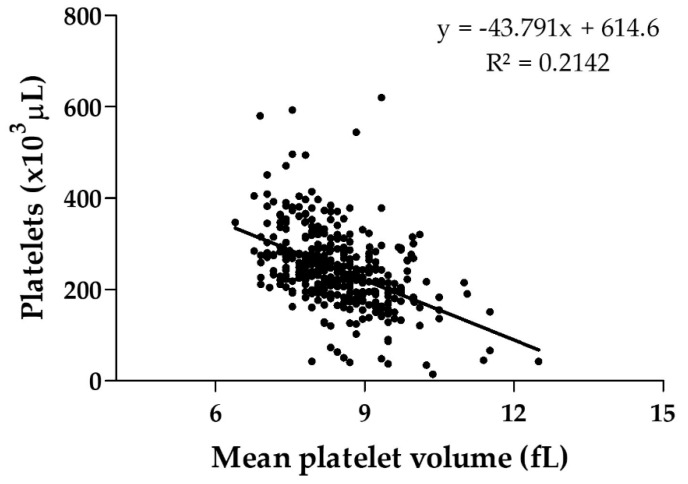
Platelet counts and mean platelet volume obtained on admission for *Bothrops* snakebite patients. Pearson correlation coeficiente (*r*) between the variables was equivalent to −0.463 (*p* < 0.001).

Median platelet count on admission of *Bothrops* snakebite patients with (217,000/µL) and without (239,500/µL) systemic bleeding during hospitalization showed significant statistical differences (*p* = 0.035) (Figure 2). Negative (inverse) correlation was found between the variables of number of platelets and mean platelet volume on admission (*r* = −0.463; *p* < 0.001) for absolute values during testing (Figure 3). 

**Figure 4:**
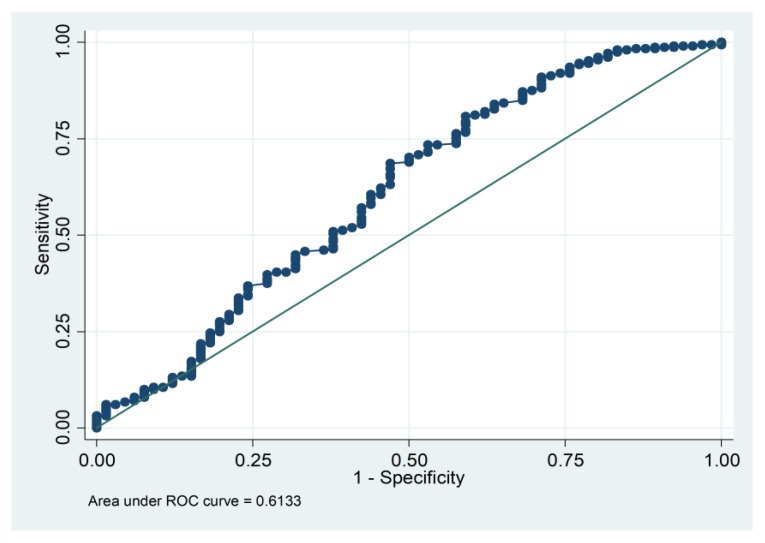
Receiver operating characteristics (ROC) curve depicting the discriminatory performance of the platelet count obtained on admission (AUROC = 0.6133; 95% CI = 0.531 to 0.696; *p* = 0.007) for systemic bleeding during hospitalization in *Bothrops* snakebite patients. AUROC, area under ROC curve; continuous line: Reference.

The area under the Receiver operating characteristics (ROC) curve showed that platelet counts obtained on admission presented a poor ability for discriminating *Bothrops* snakebite patients with systemic bleeding during hospitalization (AUROC = 0.6133; 95% CI = 0.531 to 0.696; *p* = 0.007) (Figure 4). 

**Figure 5:**
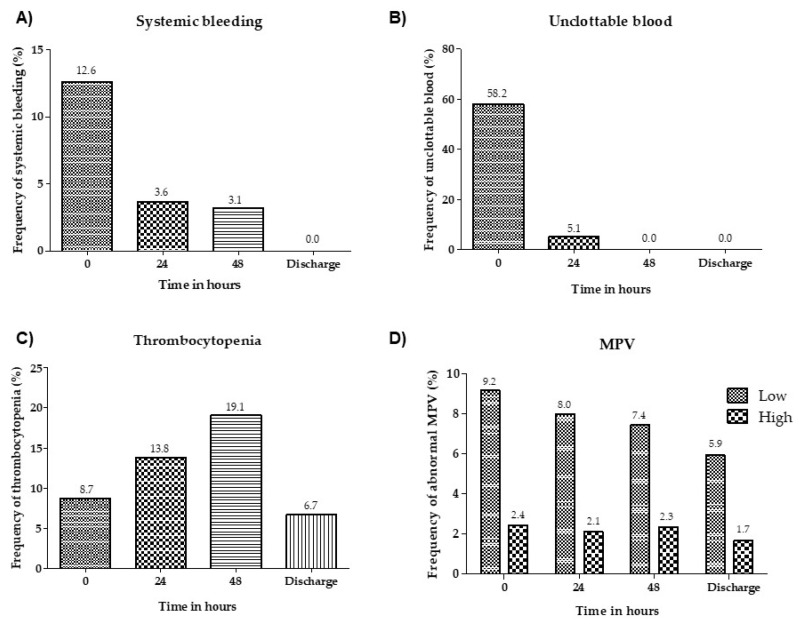
Frequency of systemic bleeding (**A**), unclottable blood (**B**), thrombocytopenia (platelet count <150,000/µL) (**C**) and abnormal mean platelet volume, low (<7.4 fL) and high (>10.4 fL) (**D**) on admission, 24 h and 48 h after administration of the specific antivenom and on discharge. MPV: Mean platelet volume.

Systemic bleeding and unclottable blood presented an improvement within 24 h in most patients after administration of the specific antivenom, while the frequency of thrombocytopenia increased in the first 48 h after antivenom administration, though it did decrease on discharge, and the frequency of abnormal mean platelet volume showed a decrease until discharge (Figure 5). No bleeding episode was recorded in patients who returned for clinical reassessment after being discharged.

### 2.3. Factors Associated with Systemic Bleeding

A total of 63 *Bothrops* snakebite patients with systemic bleeding during hospitalization were included as cases and 349 *Bothrops* snakebite patients with non-systemic bleeding were included as controls. Unclottable blood (AOR = 3.11 (95% CI = 1.53 to 6.31; *p* = 0.002)) and thrombocytopenia (AOR = 4.52 (95% CI = 2.03 to 10.09; *p* < 0.001)) on admission were independently associated with the risk of developing systemic bleeding after a *Bothrops* snakebite during hospitalization (Table 5).

## 3. Discussion

*Bothrops* snake envenomation usually results in systemic effects such as coagulation disorders and systemic bleeding [7,22]. Although systemic bleeding is a common clinical manifestation in *Bothrops* snakebites, the clinical–epidemiological and laboratorial factors associated with the development of this effect are not well known in relation to the envenomations that occur in the Amazon region. In this study, systemic bleeding was present in 15.3% of the *Bothrops* snakebite patients during hospitalization, the most common being conjunctival and gingival, in consonance with previous studies from the Brazilian Amazon [7,23,24]. However, this rate was lower than that observed in envenomations by other species of *Bothrops* in Central and South America [15,21,25], which suggests that other mechanisms may be involved in the development of systemic bleeding.

The majority of *Bothrops* snakebites occurred in males, between 11 and 30 years old, living in rural areas and were mainly unrelated to work. Most of our patients took less than 3 h to reach medical assistance. These characteristics were also observed in other studies regarding the Brazilian Amazon and other regions of Brazil [4,21,23], as well as in other countries [16,26]. However, there was no significant association between systemic bleeding and epidemiological aspects of *Bothrops* snakebites in this study, although age and time taken to reach medical assistance were independently associated with severity and mortality in other studies of snakebites conducted in the Brazilian Amazon [4].

In this study, thrombocytopenia was an infrequent event. This observation was similar to that found in another study carried out in Belém, Pará State, Brazil, where *B. atrox* also causes most of the snakebites [7]. Contrary the this, *B. jararaca* snakebites showed a higher frequency of thrombocytopenia [15,21,22,27]. Most of the cases of systemic bleeding observed in our study showed a normal platelet count on admission, irrespective of the type of systemic bleeding, except for one case of petechiae. However, although thrombocytopenia is an uncommon finding, in this study thrombocytopenia on admission was independently associated with the development of systemic bleeding during hospitalization.

Indeed, platelets participate in reactions of hemostasis such as in adhesion to the cut end of a blood vessel, spreading of adherent platelets on the exposed subendothelial surface, secretion of stored platelet constituents, and formation of platelet aggregates [28,29]. In addition, platelets interact with proteins of the coagulation through surface receptors and phospholipids [30,31]. Recently, it has been observed that platelets also have roles in immune and inflammatory processes [32,33]. In snakebites, thrombocytopenia probably results from a multifactorial etiology as venom-induced platelet aggregation [34], sequestration to the areas of damage near the site of the bite [35], and venom-induced oxidative stress leading to a drop-in platelet count by apoptosis [36]. Components isolated from the *B. atrox* venom such as thrombocytin [34], batroxostatin [37], botrocetin [38], and batroxrhagin [39] may be acting on platelets leading to thrombocytopenia or changes in platelet function.

On the other hand, a negative correlation was found between the number of platelets and mean platelet volume on patient admission, which suggests a consequence of peripheral platelet destruction in which the mean platelet volume tends to increase. Studies show large platelets are more reactive than ordinary size platelets, as measured by aggregation and total release of granular content [40]. However, the ROC curve showed that only platelet counts on admission are not a good predictor of systemic bleeding during hospitalization in this specific envenomation, indicating that other parameters are involved in the development of systemic bleeding during hospitalization.

In this study, unclottable blood on admission was observed in more than half of *Bothrops* snakebite patients. The coagulation disorders observed in *B. atrox* snakebites patients can be explained by the presence of components of the venom with thrombin-like activity, which directly hydrolyze fibrinogen in fibrin [41,42,43] and pro-coagulants, which activate coagulation factors II and X [44,45], which results in the intravascular thrombin generation. Other coagulation factors activated by components isolated from *B. atrox* venom are the factors XIII [34] and V [46]. In addition, *B. atrox* venom components that present fibrin(geno)lytic activity can also contribute to coagulopathy [47]. Unclottable blood on admission was independently associated with the development of systemic bleeding during hospitalization in this study.

We observed a significative association between thrombocytopenia and unclottable blood on admission in *Bothrops* snakebites patients who developed systemic bleeding during hospitalization. Nonetheless, the poor results found in the discriminative analysis (ROC curve) prompts us to believe that these parameters alone are not the only ones involved in the systemic bleeding phenomena shown here. This corroborates with findings regarding *B. jararaca* snakebites patients, which suggest that systemic bleeding may occur in patients with coagulable blood and thrombocytopenia and that coagulation disorders are not therefore the primary cause of systemic bleeding [27]. Similar reports have been described with *B. atrox* snakebites in Colombia [20]. 

P-III SVMP is the markedly predominant toxin of the *B. atrox* venom, which comes from the snake responsible for the majority of snakebites in the Brazilian Amazon [12]. Interestingly, a PIII-SVMP called Batroxrhagin, the major component of *B. atrox* venom, is highly similar to Jarharagin, and is able to inhibit collagen-induced platelet-aggregation, hydrolyze fibrin, and is highly hemorrhagic [39]. P-III hemorrhagic SVMPs induce hemorrhage by their accumulation at the basement membrane by binding to collagens [48,49]. P-I SVMPs, such as batroxase [47] and atroxlysin-I [50], that induce bleeding through hydrolysis of extracellular matrix components have also been isolated from *B. atrox* venom. In addition, it has been observed in *Bothrops* snakebites that haemorrhagins present in snake venom cause local haemorrhage and systemic bleeding by the direct action on components of the basement membrane of capillaries [15,51]. Serum haemorraghin levels were significantly higher in patients with clinical signs of systemic bleeding than those without in envenomation by *B. jararaca* snakes. Haemorraghin levels were also correlated inversely with platelet count in these patients [15]. In our study, high serum haemorraghin levels probably also contributed to the development of systemic bleeding in *Bothrops* snakebites in the Brazilian Amazon. However, these levels were not determined. Likewise, changes in platelet function were not evaluated.

Another aspect to be considered in this study was antivenom therapy of these *Bothrops* snakebites. *Bothrops* antigen used in Brazil to immunize horses in order to produce the antivenom consists of a mixture of venoms of *B. jararaca* (50%), *B. alternatus* (12.5%), *B. moojeni* (12.5%), *B. neuwiedi* (12.5%), and *B. jararacussu* (12.5%) (manufacturer’s data). *B. atrox* venom is not included in the immunization pool used to produce *Bothrops* antivenom. Nevertheless, the results suggest that venom-induced systemic bleeding and unclottable blood were able to be ceased in most patients after 24 h of antivenom administration. These results were also found in other studies on *Bothrops* snakebites in the Brazilian Amazon [7,19] and other regions of Brazil [22]. On the other hand, it has been noted that *Bothrops* antivenom is less effective for neutralizing factor X activation activity of *B atrox* venom when compared to prothrombin activation activity [52]. In contrast, the frequency of thrombocytopenia increased in the first 48 h after antivenom administration, though decreased on discharge, while the frequency of abnormal mean platelet volume showed a decrease on discharge. Studies propose that venom-induced oxidative stress could lead to thrombocytopenia by apoptosis [36]. In *Bothrops jararaca* and *B. jararacussu* snakebites, it was observed that *Bothrops* envenomation promotes persistent and pronounced oxidative stress in the blood of the victims up to 1 month after hospitalization [53], which could explain the thrombocytopenia observed in our study up to 48 h after antivenom administration.

In conclusion, systemic bleeding observed in *Bothrops* snakebite patients in this part of the Amazon was an important systemic effect of the envenomation. The prevalence of systemic bleeding in this study was similar to that of others from the same region, but lower than that of other *Bothrops* spp envenomations. Thrombocytopenia and unclottable blood detected on admission were independently associated with the risk of developing systemic bleeding during hospitalization, which suggests that these hemostatic disorders increase the chances of systemic bleeding. Moreover, the effect of other parameters such as some toxins components of the venom can be involved in the development of systemic bleeding. Prospective studies are needed to elucidate the pathophysiology of systemic bleeding in *Bothrops* snakebites in the Brazilian Amazon and identify the different factors involved in its development as well as on the therapeutic response of hemostatic disorders.

## 4. Materials and Methods 

### 4.1. Study Design and Data Source

This was an observational, cross-sectional study designed to assess the prevalence of and factors associated with systemic bleeding in *Bothrops* snakebite patients. Clinical–epidemiological and laboratorial data were obtained from patients’ records with clinical–epidemiological diagnosis of *Bothrops* envenomation who were attended to at the *Fundação de Medicina Tropical Dr. Heitor Vieira Dourado* (FMT-HVD), a specialist hospital for snakebites in Manaus, capital of the Amazonas State, Brazil, between August, 2013 and July, 2016. Eligible cases were those with clinical signs of *Bothrops* envenomation. Patients that underwent previous antivenom therapy in other health service centers were not included in this study. According to the Brazilian Ministry of Health Guidelines for Snakebite Diagnosis and Treatment [54], envenomation by *Bothrops* species is classified as follows: (a) Mild, characterized by pain and mild or absent local edema, mild or absent systemic bleeding, with or without change in coagulation time; (b) moderate: Characterized by pain and evident edema involving three or more segments of the affected limb, accompanied or not by systemic bleeding and coagulopathy; (c) severe: Characterized by edema involving the entire affected limb, usually accompanied by severe pain. Systemic manifestations such as hypotension, shock, oliguria/anuria, or severe bleeding are defined as a severe case, regardless of the local effect. This study was conducted in accordance with the Declaration of Helsinki, and the protocol was approved by the Ethics Committee of the FMT-HVD, Manaus, Brazil (approval number: 1.433.431, approval date: 2 March 2016). Term of commitment of use of data was signed by the researchers. 

### 4.2. Clinical–Epidemiological and Laboratorial Parameters

The clinical–epidemiological variables analyzed were gender, age (in years), geographical location of the occurrence of the snakebite, type of area where the snakebite occurred (rural or urban), work-related bite, occupation, time taken to reach medical assistance (in hours), anatomical region of the bite, pre-hospital treatment (use of topical or oral medicines, use of tourniquet and other procedures), *Bothrops* bite confirmation by snake, local and systemic manifestations, clinical severity of envenomation (mild, moderate, or severe according to the Brazilian Health Ministry guidelines), presence of comorbidities, time spent in hospital (in days), and outcome (discharge or death). Analyzed laboratory variables were clotting time (in minutes), hemoglobin (mg/dL), hematocrit (%), platelet count (number/μL), and mean platelet volume (fL). The snakes were identified by a trained biologist at the FMT-HVD. All variables were checked by two independent researchers before analysis and further investigated for possible association with systemic bleeding as a dependent variable.

Snakebite-induced coagulopathy was evaluated by a modification of the Lee–White clotting time (LWCT) [55] which is recommended by the Brazilian Ministry of Health. With the use a plastic unlubricated syringe, 1 mL of venous blood was collected and placed into a new glass tube (13 × 75 mm) without any anticoagulants at 37 °C. Using a stopwatch, the timing started as soon as the blood was drawn into the tube. The glass tube was left undisturbed for 5 min and then checked for clots every following minute by gently tilting the tube. Unclottable blood was defined when the blood was not clotted until 30 min. The sensitivity of the LWCT performed in routine clinical settings for the diagnosis of hypofibrinogenemia is 78% [56]. Thrombocytopenia was defined by a platelet count below 150,000/μL. Low hemoglobin was defined when values were lower than 12.5 g/dL. Low hematocrit was defined when values were below 36%. Low and high mean platelet volume was defined when values were below 7.4 fL and above 10.4 fL, respectively. In order to identify clinical–epidemiological and laboratorial variables collected on admission associated with systemic bleeding, patients evolving to systemic bleeding on admission or during hospitalization were classified as a case, and one with non-systemic bleeding was included as a control. All patients were asked to return to the hospital for clinical reassessment in case of complications at any time, or on day 7 after discharge.

### 4.3. Statistical Analysis

A database and descriptive analyses were produced using Microsoft Excel Office 365 software which was fed information by two independent individuals. Statistical analyses were made using the software STATA statistical package version 13 (Stata Corp, College Station, TX, USA) and Graphpad Prism version 5 (Graphpad Software, Inc., San Diego, CA, USA). The Mann–Whitney test was used for comparison of medians. Differences in results were considered statistically significant when *p* < 0.05. The Pearson correlation coefficient was calculated to measure the degree of association between the platelet count and mean platelet volume on admission. The performance of platelet counts on admission to predict systemic bleeding during hospitalization in *Bothrops* snakebite patients was assessed using ROC curve.

Proportions of systemic bleeding during hospitalization were compared by Chi-square test (corrected by Fisher’ test if necessary); differences were considered statistically significant for *p* < 0.05. The crude Odds Ratio (OR) with its respective 95% Confidence Interval (95% CI) was determined considering systemic bleeding during hospitalization as the dependent variable. Logistic regression was used for the multivariable analyses and the adjusted Odds Ratios with 95% CI were also calculated. All variables associated with the outcome at a significance level of *p* < 0.20 in the univariate analysis were included in the multivariable analysis. Statistical significance was considered if *p* < 0.05 in the Hosmer–Lemeshow goodness-of-fit test. 

## Figures and Tables

**Table 1:** Background information of *Bothrops* snakebite patients obtained on admission.

Characteristics (*n*; Completeness)	Number	%
Gender (*n* = 412; 100%)		
Male:female	322:90	
Age group in years (*n* = 412; 100%)		
0–10	41	10.0
11–20	83	20.2
21–30	83	20.2
31–40	60	14.6
41–50	54	13.1
51–60	51	12.4
>60	40	9.7
Geographic location of the snakebite incident (*n* = 411; 99.76%)		
Capital (municipality of Manaus)	236	57.4
Municipalities of the interior of the Amazonas State	171	41.6
Other States	4	1.0
Area of occurrence (*n* = 410; 99.52%)		
Rural	365	89.0
Urban	45	11.0
Work-related bite (*n* = 332; 80.58%)		
Yes	132	39.8
Occupation (*n* = 171; 41.51%)		
Rural worker	85	49.7
Other	86	50.3
Time-taken to reach to medical assistance (h) (*n* = 410; 99.52%)		
0–3	245	59.8
4–6	85	20.7
>6	80	19.5
Anatomical location of bite (*n* = 412; 100%)		
Foot	297	72.1
Lower Leg	59	14.3
Thigh	4	1.0
Hand	49	11.9
Arm	3	0.7
Pre-hospital treatment		
Tourniquets (*n* = 314; 76.21%)	56	17.8
Local incisions (*n* = 313; 75.97%)	17	5.4
Use of topical/oral medicines (*n* = 314; 76.21%)	167	53.2
*Bothrops* bite confirmation (*n* = 412; 100.0%)		
Confirmed by snake identification	135	32.8
Clinical-epidemiological diagnosis	277	67.2

**Table 2:** Clinical characteristics and comorbidities of *Bothrops* snakebite patients obtained on admission.

Variable	Number	%
Local manifestations (*n* = 412; 100%)		
Pain	385	93.9
Swelling	382	93.4
Bleeding from fang punctures	120	29.3
Redness	120	29.1
Ecchymosis	36	8.7
Paresthesia	35	8.5
Haematoma	8	1.9
Blistering	7	1.7
Regional lymphadenopathy	4	1.0
Systemic alterations related to hemostasis		
Unclottable blood (*n* = 386; 93.69%)	224	58.2
Bleeding (*n* = 412; 100%)	52	12.6
Trombocytopenia (*n* = 379; 91.99%)	33	8.7
Other systemic manifestations (*n* = 412; 100%)		
Headache	47	11.4
Nausea	44	10.7
Vomiting	40	9.7
Blurred vision	14	3.4
Abdominal pain	7	1.7
Diarrhoea	5	1.2
Clinical severity of envenomation (*n* = 404; 98.06%)		
Mild	112	27.7
Moderate	219	54.2
Severe	73	18.1
Comorbidities (*n* = 313; 75.97%)		
Arterial hypertension	29	9.3
Diabetes	8	2.6
Hepatopathy	4	1.3
Cardiopathy	3	1.0
HIV/Aids	1	0.3
Time spent in hospital (days)		
Mean (range) (*n* = 405, 98.30%)	7	(1–41)
Outcome (*n* = 411; 99.76%)		
Discharged	411	100.0

**Table 3:** Systemic bleeding of *Bothrops* snakebite patients during hospitalization.

Description	Number	%
**Systemic bleeding (*n* = 412; 100%)**	63	15.3
Conjunctival	27	6.7
Gingival	26	6.3
Macrohematuria	18	4.4
Haematemesis	12	2.9
Haemoptysis	10	2.4
Epistaxis	10	2.4
Ecchymosis	7	1.7
Enterorrhage	5	1.2
Metrorrhagia	3	0.7
Petechiae	1	0.2
From recent wound	1	0.2

**Table 4:** Systemic bleeding of *Bothrops* snakebite patients during hospitalization versus platelet count on admission.

	Platelet Count		
	<50 × 10³/µL *n* (%)	50–150 × 10³/µL *n* (%)	>150 × 10³/µL *n* (%)
Systemic bleeding (*n* = 55; 100%)	5 (9.1)	8 (14.5)	42 (76.4)
Conjunctival	0 (0.0)	2 (8.3)	22 (91.7)
Gingival	2 (8.3)	4 (16.7)	18 (75.0)
Macrohematuria	1 (6.3)	4 (25.0)	11 (68.7)
Haematemesis	2 (20.0)	2 (20.0)	6 (60.0)
Haemoptysis	1 (11.1)	3 (33.3)	5 (55.6)
Epistaxis	0 (0.0)	1 (11.1)	8 (88.9)
Ecchymosis	0 (0.0)	0 (0.0)	5 (100.0)
Enterorrhage	0 (0.0)	1 (25.0)	3 (75.0)
Metrorrhagia	0 (0.0)	1 (33.3)	2 (66.7)
Petechiae	1 (100.0)	0 (0.0)	0 (0.0)
From recent wound	0 (0.0)	0 (0.0)	1 (100.0)

**Table 5:** Parameters obtained on admission associated with systemic bleeding (SB) of *Bothrops* snakebite patients during hospitalization.

Description	SB (*n*)	%	Non-SB (*n*)	%	Crude OR (IC 95%)	*p*	Adjusted OR (IC 95%)	*p*
Sex								
Female	15	16.7	75	83.3	0.88 (0.46–1.65)	0.682		
Male	48	14.9	274	85.1				
Age group in years								
0–10	6	14.6	35	85.4	1			
10–20	14	16.9	69	83.1	1.18 (0.42–3.35)	0.751		
20–30	10	12.1	73	87.9	0.79 (0.27–2.37)	0.687		
30–40	7	11.7	53	88.3	0.77 (0.24–2.48)	0.662		
40–50	12	22.2	42	77.8	1.66 (0.57–4.90)	0.353		
50–60	8	15.7	43	84.3	1.08 (0.34–3.42)	0.889		
>60	6	15.0	34	85.0	1.03 (0.30–3.51)	0.963		
Area of occurrence								
Urban	3	6.7	42	93.3	2.75 (0.83–9.18)	0.099	2.28 (0.66–7.84)	0.190
Rural	60	16.4	305	83.6				
Time-taken to reach to medical assistance (h)								
0–3	30	12.2	215	87.8	1		1	
4–6	15	17.6	70	82.4	1.54 (0.78–3.02)	0.214	0.83 (0.37–1.85)	0.644
>6	18	22.5	62	77.5	2.08 (1.08–3.98)	0.027	1.83 (0.88–3.79)	0.103
Pre-hospital treatment								
Yes	33	14.4	197	85.6	0.92 (0.46–1.84)	0.802		
No	13	15.5	71	84.5				
Comorbidities								
Yes	8	17.8	37	82.2	1.24 (0.51–3.01)	0.637		
No	22	14.9	126	85.1				
Unclottable blood								
Yes	48	21.4	176	78.6	3.72 (1.86–7.42)	<0.001	3.11 (1.53–6.31)	0.002 *
No	11	6.8	150	93.2				
Low hemoglobin								
Yes	11	17.7	51	82.3	1.29 (0.63–2.67)	0.486		
No	44	14.3	264	85.7				
Low hematocrit								
Yes	5	11.1	40	88.9	0.68 (0.26–1.81)	0.444		
No	50	15.5	273	84.5				
Thrombocytopenia								
Yes	13	39.4	20	60.6	4.71 (2.18–10.15)	<0.001	4.52 (2.03–10.09)	<0.001 *
No	42	12.1	304	87.9				
Mean platelet volume								
Low	5	14.7	29	85.3	1		1	
Normal	44	13.5	282	86.5	0.91 (0.33–2.46)	0.845	0.70 (0.24–2.00)	0.503
High	4	44.4	5	55.6	4.64 (0.92–23.48)	0.064	3.46 (0.56–21.55)	0.182

* *p* < 0.05 was considered significant. Thrombocytopenia: platelet count <150,000/μL; Low hemoglobin: <12.5 g/dL; Low hematocrit: <36%: Low mean platelet volume: <7.4 fL; High mean platelet volume: >10.4 fL.
